# Notes and News

**Published:** 1892-09-24

**Authors:** 


### Notes and News.

Collected and Communicated.

A magnificent sanatorium has just been opened at Leysin, in Switzerland. The site is all that could be desired. The building, which is erected on high ground and upon a dry soil, is protected from both north and east winds. The climate of Leysin is most suitable to invalids. The interior of the building has been provided with every comfort, and arranged according to hygienic principles. The sanitorium has been placed under the management of Dr. Lauth, formerly medical officer at a Paris hospital.

At the meeting of the Metropolitan Asylums Board on Saturday, the Local Government Board received a sorry character. The fever hospitals are full, whilst large numbers of applications for admission are refused daily, scarlet fever showing but little diminution. The Local Government Board have not been allowed to remain ignorant of the exigencies of the case; they cannot be blind to the fatal policy of procrastination under such conditions, and yet nothing has been done. Penny wise, pound foolish is the least that can be said of such policy as theirs. Something very much stronger will no doubt be said in many quarters before long. The Asylums Board, it appears, are powerless to remedy what the higher powers leave undone, and the public suffer in consequence.

At the same meeting of the Asylums Board which revealed so unsatisfactory a state of affairs in regard to the fever hospitals, a most satisfactory statement of the actions and organisation of the Cholera Committee was given. Here forethought and efficiency show up in striking contrast to the other picture. The ambulance stations have all been provided with litters for the conveyance of the sick, the extra service of carriers having also been provided for. A complete list has been drawn up showing the locality and number of beds ready for occupation throughout the metropolis; and in a word, the committee have an excellent organization ready for work if necessary. Reasonable hopes are entertained that this year, at any rate, we are to be spared an epidemic; but the confidence inspired by such a state of readiness would in itself render the disease less likely to gain a footing.

In view of removing the prevailing difficulty of locating isolation hospitals, a facetious contemporary suggests an aerial hospital, suspended by means of a balloon. The idea is very suggestive.

The war against the snowball system, which has been waging for some time in the *Times*, must have some good result, since it has exposed the evils of the system exemplified in the strongest degree in the case of the Alexandra Hospital Snowball. Mrs. Marsh, the author of the snowball in this instance, admits to having received no less than £964 in stamps, all of which she has not yet succeeded in converting into money. Mrs. Marsh herself acknowledges the evil of the system over which the starter has no control, and it is to be hoped that the public will not be so mistaken as to join in such methods of obtaining money for any cause in the future.

The admission of lady students at the Glasgow Royal Infirmary has certainly not proved successful so far. It appears from a subscriber's letter to a local paper that not only have the ladies taken possession of those wards which were especially reserved for them, to the exclusion of male students, but they have joined with the men in various classes, which has so provoked their ire that they have refused to attend any longer if the ladies continue to do so. The reasons given for this step may be as disinterested as they appear, or they may not, but under any circumstances those who are appealing on behalf of the lady students cannot have a very strong case so long as Queen Margaret's College, Glasgow, announces itself a medical school for women. The professors and lecturers are the same as those of the University, whilst the curriculum, regulations, and fees profess to be also identical. Provided practical experience is forthcoming, we imagine the ladies will have to be content for the present, and try again another day.

Whilst the subject of lady students at Glasgow is still on the tapis, the same question has arisen in Cork, application for admission to the clinical instruction at the South Infirmary having been made by a lady student. A most interesting debate arose from the application at the meeting of the Board of Trustees of the infirmary. Professor Hartog, of the Queen's College, came to second the lady's application in person. In his speech, Professor Hartog said that the experiment was no new one in Ireland. It was carried out with perfect success at Belfast and Dublin. The present limitations of instruction open to female students made it impossible for them to complete their medical education in Cork. He pointed out that the objection usually given to the study of medicine for women, side by side, with male students, held good in the cases of nurses, on account of whom, however, the question was not raised. Perhaps the professor's strongest point was the fact that the students of the South Infirmary themselves join in the application. Ireland has always been foremost in its chivalrous conduct towards women. Here we have another example, offering a marked contrast to the attitude of the Scottish students. The days when women were first admitted to medical studies in Edinburgh are not likely soon to be forgotten. The behaviour of the students at that time cast a slur on their manhood, and of which they, doubtless, in maturer years, are heartily ashamed, and whether we agree with the cause they advocate or not, we are bound to admit that the chivalrous and more generous-minded Irish student claims our admiration.

